# Global trends and forecasts of breast cancer incidence and deaths

**DOI:** 10.1038/s41597-023-02253-5

**Published:** 2023-05-27

**Authors:** Yuyan Xu, Maoyuan Gong, Yue Wang, Yang Yang, Shu Liu, Qibing Zeng

**Affiliations:** 1 Guizhou Medical University, the Key Laboratory of Environmental Pollution Monitoring and Disease Control, Ministry of Education & Guizhou Provincial Engineering Research Center of Ecological Food Innovation & School of Public Health, Guiyang, 550025 China; 2 The Affiliated Hospital of Guizhou Medical University, Department of Breast Surgery, Guiyang, 550004 China

**Keywords:** Cancer epidemiology, Risk factors

## Abstract

Breast cancer (BC) is one of the major public health challenges worldwide. Studies that address the new evidence on trends of BC are of great importance for preventing and controlling the occurrence and development of diseases and improving health. The aim of this study was to analyze the outcomes for the global burden of disease (GBD), incidence, deaths, and risk factors for BC from 1990 to 2019, and predict the GBD of BC until 2050 to inform global BC control planning efforts. In this study, the results show that the regions with low levels of socio-demographic index (SDI) will have the largest disease burden of BC in the future. The leading global risk factor for death attributable to BC in 2019 was metabolic risks, followed by behavioral risks. This study supports the worldwide urgent need for comprehensive cancer prevention and control strategies to reduce exposure, early screening, and improve treatment to effectively reduce the GBD of BC.

## Introduction

Breast cancer (BC) is a most common malignant tumor, and its global burden of disease (GBD) has become one of the important factors that endanger the health of the world population, especially the health of women^1^. The global BC statistics report shows that in 2020, there will be 2.261 million new cases and 685,000 deaths worldwide, and BC has become the number one malignant tumor in the world^2^. Although some cancer cases cannot be prevented, governments can develop a range of health interventions to minimize exposure to known cancer risk factors, such as environmental factors, lifestyle behaviors, dietary habits, metabolic factors, *etc*^3^. Therefore, understanding the relative contributions of modifiable risk factors to the GBD of BC and their long-term trends is critical to inform local and global cancer control efforts.

Global Health Data Exchange provides two important research tools (GBD Comparison Tool and GBD Results Tool) that have been open sourced to quantify GBD, assessing GBD by age group, sex and time (1990 to 2019) in countries around the world), attributed to a wide range of modifiable risk factors^4^. GBD 2019 is the latest iteration of the GBD study, which provides an opportunity to assess the global cancer burden attributable to risk factors. Previous studies assessed the global, regional, and national burden of breast cancer until 2017^5–9^. A recent study^10^ evaluated the GBD of female BC from 1990 to 2019 and predicted the GBD of female BC in 2035. However, these studies know little about the global cancer burden attributable to metabolic, behavioral, diet, physical activity factors and its more longer-term future forecasts to 2050.

In this study, we report for the first time the GBD for BC attributable to a comprehensive inventory of metabolism, behavior, diet, and physical activity from 1990 to 2019, using breast cancer incidence, deaths, and risk factor results. Furthermore, this study provides a new perspective on the attributable cancer burden by estimating the risk-attributable cancer burden at global levels using incidence and deaths.

## Results

### Global burden of disease and temporal trends of breast cancer

To assess the global GBD and changing trends of BC, the incident cases, death cases and ASR of BC in 1990 and 2019 were calculated, and the estimated annual percentage change (EAPC) was used to demonstrate the temporal trends from 1990 to 2019. The global GBD and temporal trends of BC are presented in Supplementary Tables 1 and 2. Globally, the incident cases of BC increased from 876,990 in 1990 to 2,002,350 in 2019, and the EAPC for incidence increased by an average 0.33% per year. Although the death cases of BC in 2019 is higher than in 1990 worldwide, the EAPC for deaths decreased by an average 0.56% per year. In terms of gender, the number of cases and ASR of women are higher than that of men, regardless of morbidity and death. However, it is worth noting that the EAPC for incidence in men increased by an average 0.91% per year, which is higher than the woman with 0.36%. And the EAPC for deaths in different gender population both gradually decreased. Compared with other SDI regions, the incident cases, death cases and ASR of BC in high SDI regions were at a higher level. However, it is exciting to note that the EAPC for incidence began to decline in high SDI regions, and the EAPC for deaths also decreased the most in this group. In the other hand, we also observed a fast increase in the EAPC for incidence in the middle SDI regions and the EAPC for deaths in the low SDI regions. Further observation of the GBD and temporal trends of 21 GBD regions found that the highest incident cases and ASR for incidence of BC is in East Asia region, and the largest decline for EAPC is in Central Asia region. Moreover, Western Sub-Saharan Africa is the only region where EAPC for incidence continues to grow. Western Europe, Oceania and High-income North America are the region with higher breast cancer deaths in 1990, but by 2019, only the Oceania region was found to still be at relatively high levels. The EAPC for deaths in the Western Sub-Saharan Africa region increased fast, but High-income North America, Australasia and Western Europe regions decreased more obviously.

**Fig. 1:**
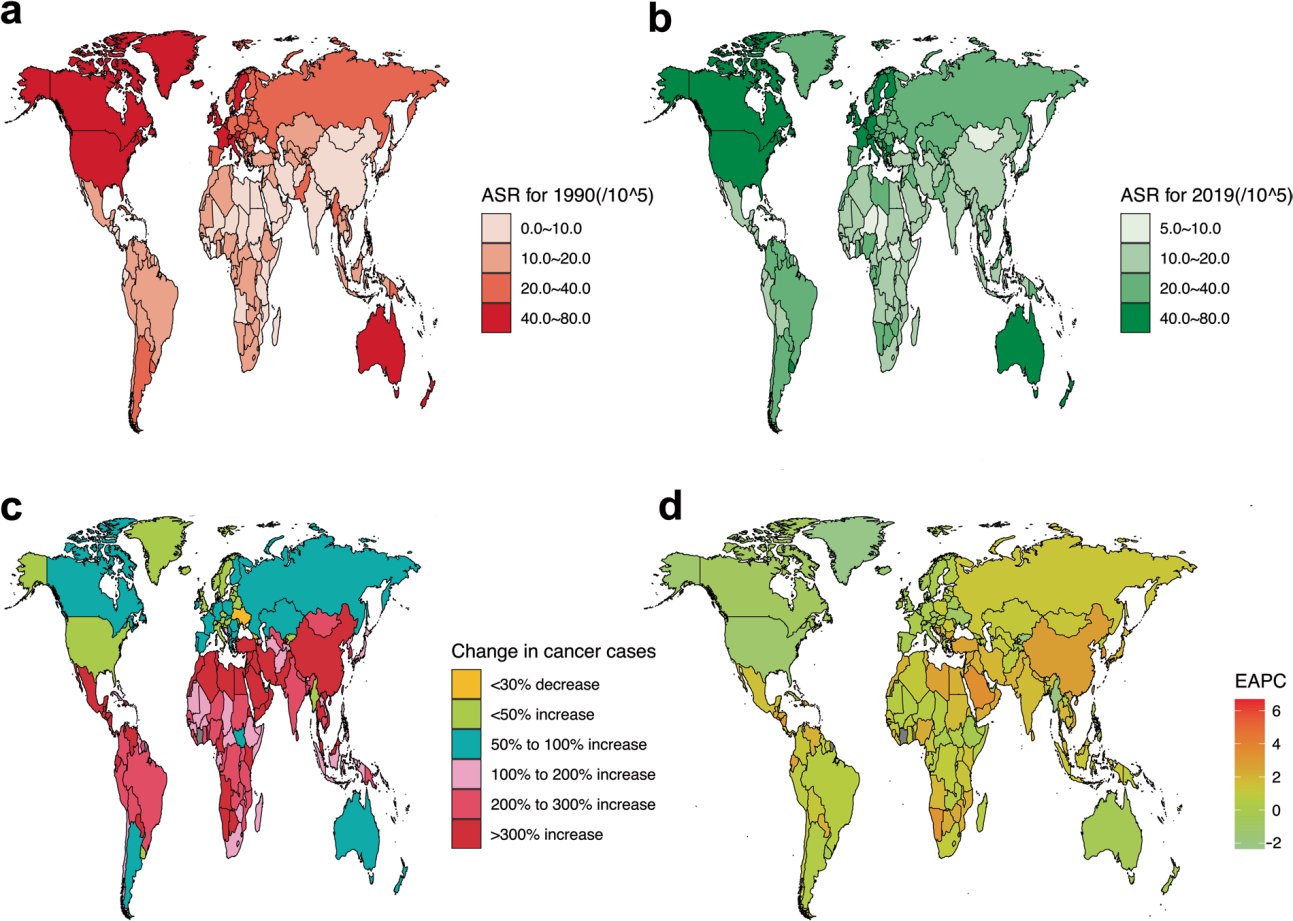
Global GBD and temporal trends of BC incidence in 204 countries or territories. ASR: age standardized rate; BC: breast cancer; EAPC: estimated annual percentage change; GBD: global burden of disease. (**a**) The ASR per 100,000 people in 1990; (**b**) The ASR per 100,000 people in 2019; (**c**) The change in cancer cases; (**d**) EAPC in different countries or territories.

**Fig. 2:**
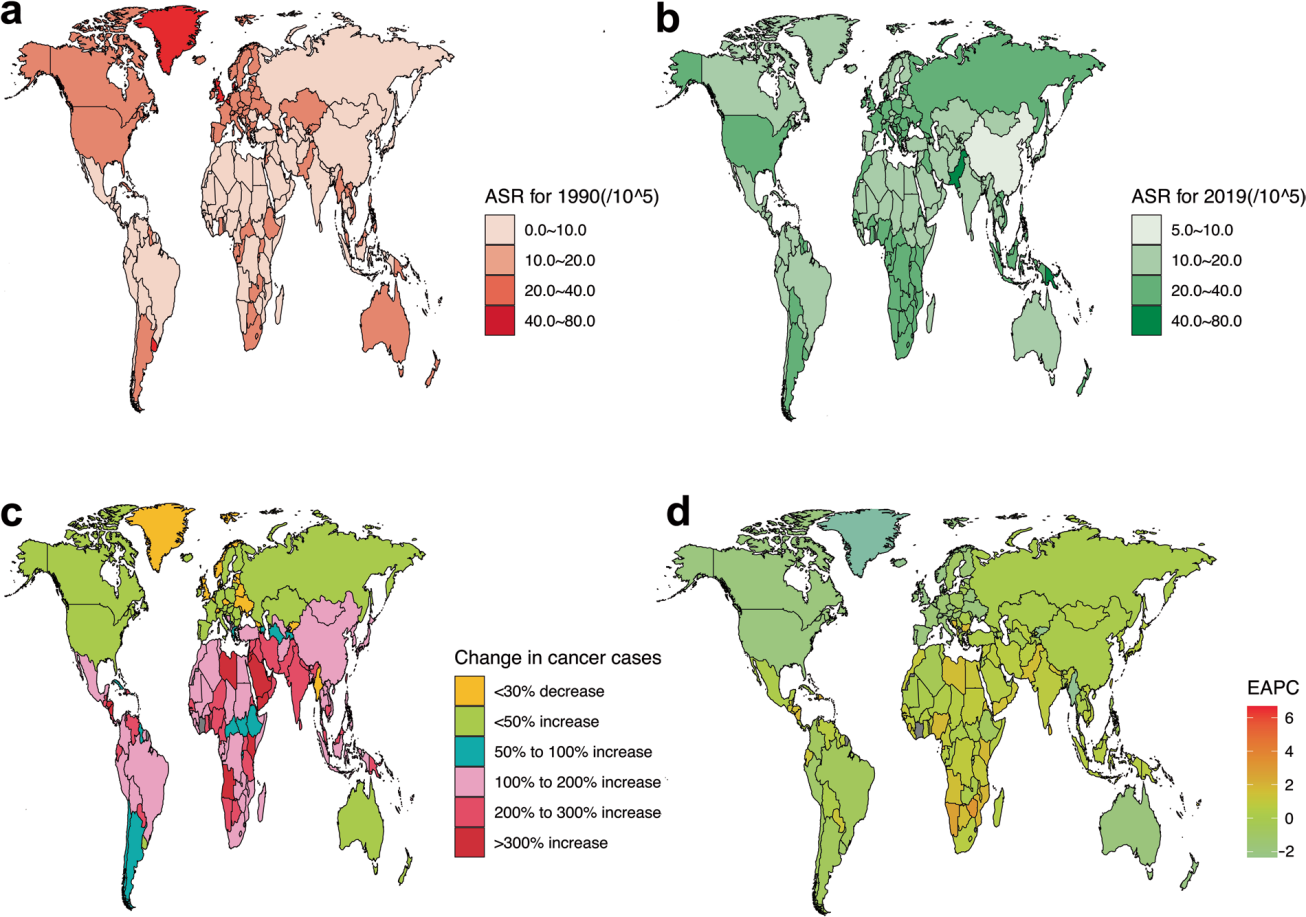
Global GBD and temporal trends of BC deaths in 204 countries or territories. ASR: age standardized rate; BC: breast cancer; EAPC: estimated annual percentage change; GBD: global burden of disease. (**a**) The ASR per 100,000 people in 1990; (**b**) The ASR per 100,000 people in 2019; (**c**) The change in cancer cases; (**d**) EAPC in different countries or territories.

Figures 1 and 2 show the GBD of BC incidence and mortality for 204 countries and territories. As shown, the countries with the highest incidence and deaths of ASR in 1990 were concentrated in high-income countries (Figs. 1a and 2a). However, the top 2 countries with the highest incidence and deaths of ASR in 2019 are not high-income countries, such as Lebanon and Solomon Islands with the highest incidence (Fig. 1b) and Pakistan and Solomon Islands with the highest death (Fig. 2b). Subsequently, we further analyzed global changes in cancer case (Figs. 1c and 2c) and EAPC (Figs. 1d and 2d) to better indicate temporal trends in GBD. From the perspective of changes in cancer cases, only 2 countries have seen a decline in incidence, while 72 countries have seen a decline in deaths. Among them, 52 countries with an increase for incidence of more than 300%, but only 3 countries had an increase for deaths of more than 300%, and the largest increase was both in the Solomon Islands.

Supplementary Fig. 1 combines EAPC for incidence and deaths data in a hierarchical cluster analysis to identify countries with similar annual growth rates in incidence and deaths. As shown in the multimedia appendices, 35 countries (or territories) were cluster into the significant increase group, including the Northern Mariana Islands, Taiwan (Province of China), Netherlands, Germany, Viet Nam, Gambia, *etc*. A total of 39 countries (or territories) were categorized into the minor increase group, including United States of America, United Kingdom, Pakistan, Canada, *etc*. Another 120 countries (or territories) were grouped into the remained stable or minor decrease group, including China, Japan, France, Mexico, and Solomon Islands. The remaining 10 countries (or territories) were categorized into the significant decrease group, including Turkmenistan, Uzbekistan, Puerto Rico, Kazakhstan, Bahrain, Colombia, Singapore, Maldives, Chile.

### Global burden of disease of breast cancer attributable to risk factors

**Fig. 3:**
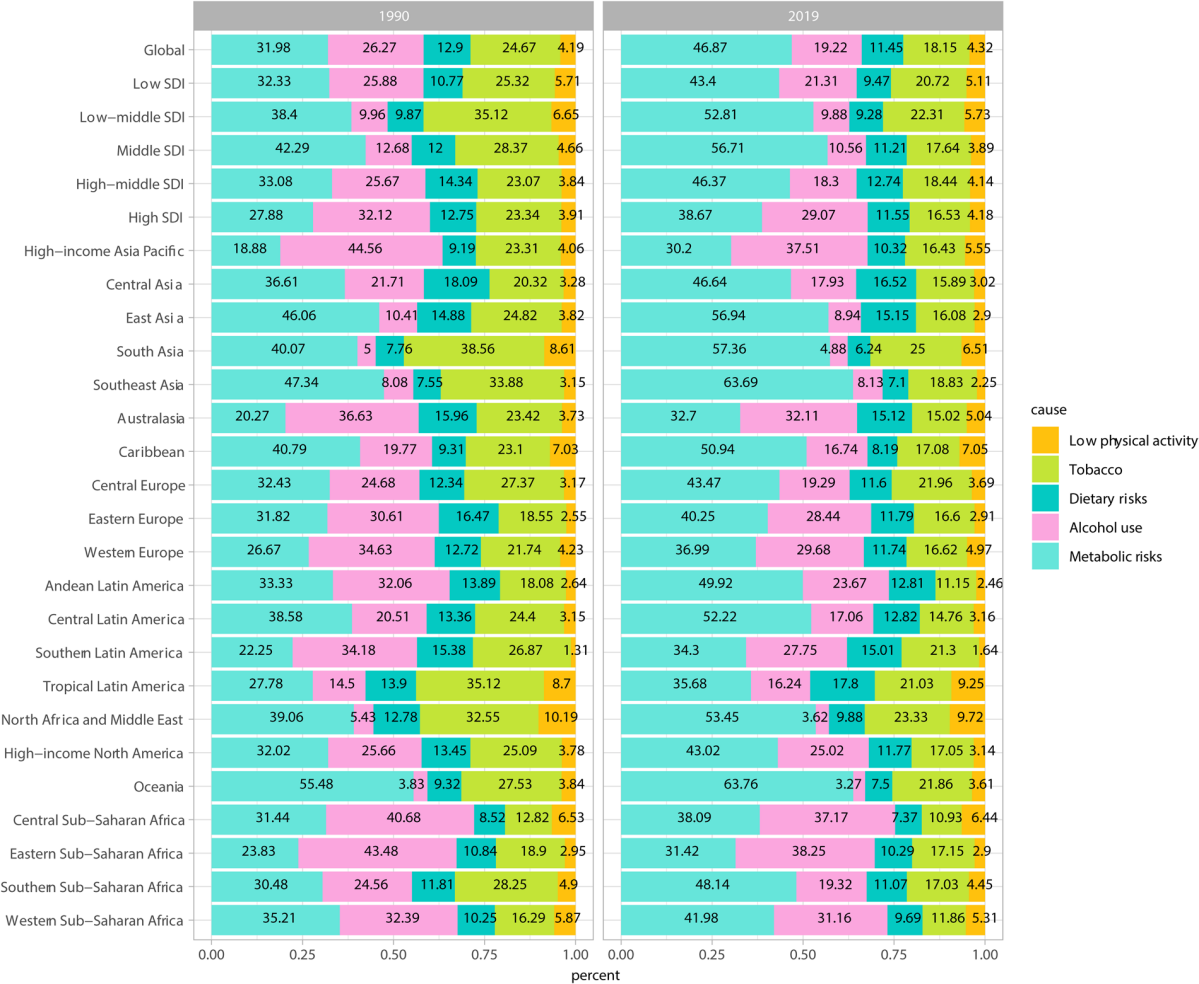
GBD of BC attributable to risk factors in 1990 and 2019. BC: breast cancer; GBD: global burden of disease; SDI, socio-demographic index.

The results of GBD of BC attributable to the risk factors were shown in Fig. 3 and Supplementary Figs. 2 and 3. As revealed in the Figure, the leading risk factor in terms of attributable BC deaths was metabolic risks worldwide, which accounted for 31.98% in 1990, and has a gradual increasing trend in 2019, accounting for 46.87%. Alcohol use, tobacco, dietary risks, and low physical activity were the next greatest risk factors. The percentage of BC deaths due to metabolic risks was significantly heterogeneous all over the world, with the highest percentage observed in Oceania region (55.48% in 1990 and 63.76% in 2019), followed by Southeast Asia region (47.34% in 1990 and 63.69% in 2019). The largest increase in the percentage for BC deaths due to the metabolic risks from 1990 to 2019 are Southern Sub-Saharan Africa (17.66%), South Asia (17.29%), Andean Latin America (16.59%), Southeast Asia (16.35%) regions. At the same time, we also observed a gradual decrease in the percentage of BC deaths due to behavioral risks such as such as alcohol use and tobacco. Dietary risks and low physical activity have remained relatively stable over the past 20 years. When we assessed the time trends of attributable risk factors at the SDI level, we found that the most increase in the percentage for BC deaths due to the metabolic risks from 1990 to 2019 are in the middle (14.42%), low-middle (14.41%) and middle-high (13.29%) SDI areas. Multimedia Appendix 2 shows the two metabolic risks attributable to breast cancer death. As shown in the figure, the global and low, middle-low and high SDI regions accounted for half and half percentage of BC deaths due to high fasting plasma glucose and high body mass index, but the fasting plasma glucose in the middle, middle-high SDI region was very high, accounting for 70.10%~ 84.62%. Furthermore, the proportion of low, low-middle, and middle SDI areas attributed to the high body mass index is increasing, especially in the low-middle SDI areas, from 42.65% in 1990 to 54.70% in 2019.

### Factors influencing the estimated annual percentage change in the global burden of disease

**Fig. 4:**
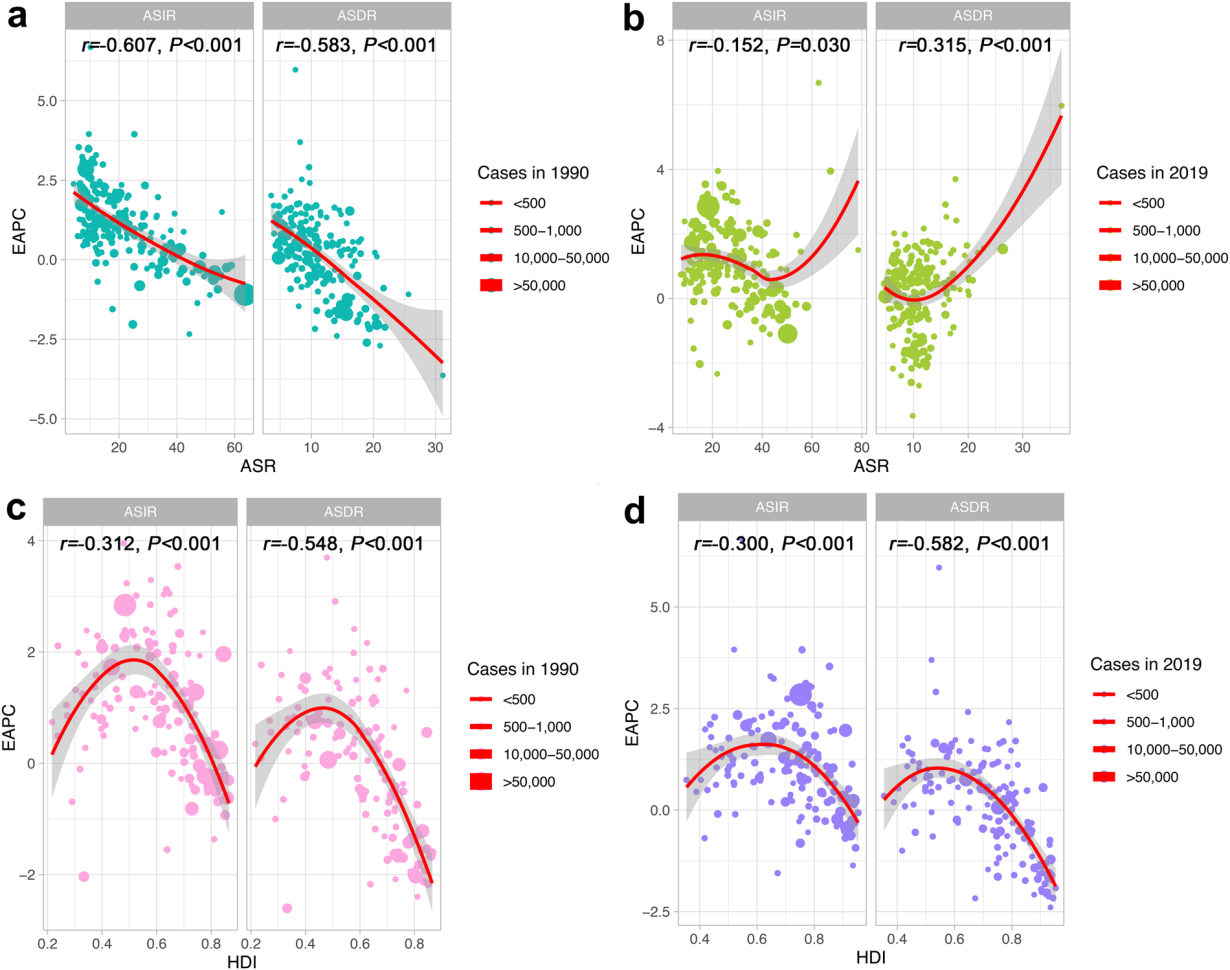
Factors Influencing EAPC in the GBD. ASR: age standardized rate; ASIR: age standardized incidence rate; ASDR: age standardized death rate; BC: breast cancer; EAPC: estimated annual percentage change; GBD: global burden of disease; HDI: human development index. (**a**) The correlation between EAPC and ASR in 1990. (**b**) The correlation between EAPC and ASR in 2019. (**c**) The correlation between EAPC and HDI in 1990. (**d**) The correlation between EAPC and HDI in 2019.

To better explain GBD in BC, we analyzed influencing factors that may affect EAPC, including ASIR, ASDR, and HDI (which can be used as an indicator of the level and availability of medical care in each country) (Fig. 4). As illustrated in the Figs. 4a,b, a significant negative correlation was found between EAPC and ASIR, ASDR in 1990 (*r* = −0.607, −0.583; *P all* < 0.001). In contrast, this negative correlation in 2019 gradually weakened or disappeared. EAPC had a weak negative correlation with ASIR (*r* = −0.152; *P* = 0.030) in 2019, but a positive correlation with ASDR (*r* = 0.315; *P < *0.001). Figure 4c,d show the correlation between the EAPC and HDI. As revealed in the figure, whether in 1990 or 2019, the relationship between EAPC and HDI is not a simple linear correlation, on the contrary it is more like a “parabola”. when the HDI was limited to below 0.50 in 1990 or 0.55 in 2019, a significant positive correlation was found between EAPC for incidence and deaths and HDI. In contrast, for a HDI above 0.50 in 1990 or 0.55 in 2019, the positive association gradually disappeared, and EAPC for incidence and deaths has a significant negative correlation with HDI in 1990 (*r* = −0.312, −0.548; *P all* < 0.001) and 2019 (*r* = −0.300, −0.582; *P all* < 0.001).

### Future forecasts of global burden of disease in breast cancer

**Fig. 5:**
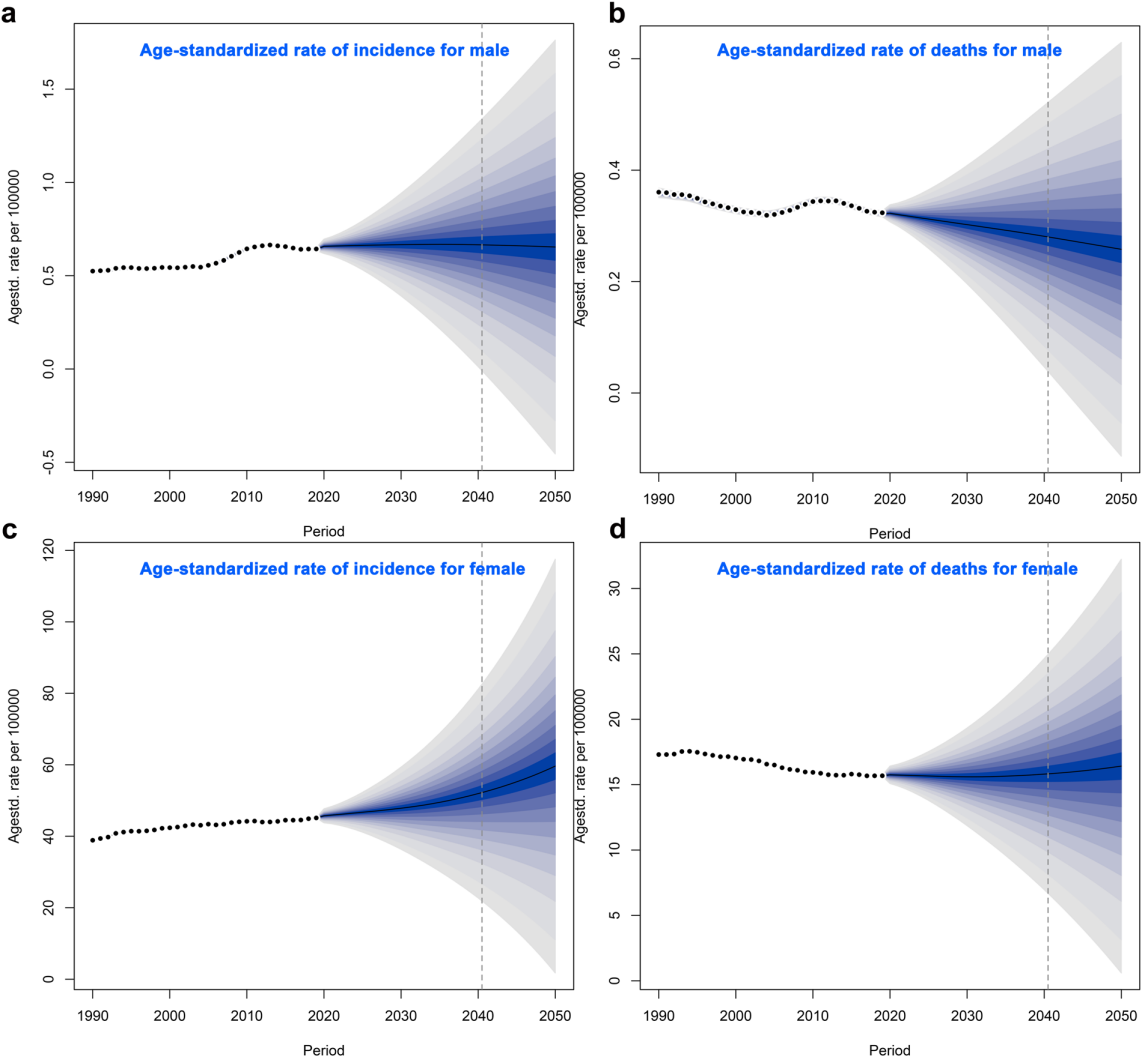
Future Forecasts of GBD in BC. ASIR: age standardized incidence rate; ASDR: age standardized death rate; BC: breast cancer. (**a**) The ASIR per 100,000 for male; (**b**) The ASDR per 100,000 for male; (**c**) The ASIR per 100,000 for female; (**d**) The ASDR per 100,000 for female.

Figure 5 show the future forecasts of GBD in BC. As illustrated in the Fig. 5a,b, the ASR of BC incidence in the world will gradually increase. It is estimated that by 2050, the ASR of BC incidence in female will be 59.63 per 100,000, an increase of 32.13% compared with 2019; the ASR of BC incidence in male will be 0.65 per 100,000, an increase of 1.74% compared with 2019. Subsequently, we also estimated the ASR of global BC deaths from 2020 to 2050 (Fig. 5c,d). Over time, the ASR of BC death in female increased slightly, but the ASR of BC deaths in male gradually decreased. It is estimated that by 2050, the ASR of female BC deaths will be 16.42/100,000, an increase of 4.69% compared with 2019; the ASR of BC deaths in male will be 0.26 cases per 100,000, a decrease of 19.84% compared with 2019. According to the United Nations world population forecast data, there will be 4,781,849 incident cases (4,714,393 women and 67,456 men) and 1,503,694 death cases (1,481,463 women and 22,231 men) of BC in the world in 2050.

## Discussion

### Breast cancer incidence burden

Our analysis found that the global incident cases of BC increased from 876,990 in 1990 to 2,002,350 in 2019, a total increase of 1.28 times. This is an increase of another 5 percentage points compared to the 2017 GBD data for BC^11^, which showed that the global incident cases of BC increased by 123% between 1990 and 2017. Although we have seen a sharp increase in the BC incident cases worldwide in the past 20 years, the ASR has not shown a trend of rapid growth. And this slow growth trend is also confirmed by our EAPC results and other study^11^, which also found that from 1990 to 2017, the incidence of breast cancer worldwide increased by 123%, but the change in ASR was not obvious. Previous study^12^ has found that the changes in the number of BC cases are largely attributable to population growth and aging. This seems to explain the findings in this study well, since our study only found a significant increase in the incident cases of BC, not ASR. It suggests that reducing the global population may be one of the key factors in reducing BC incidence.

For the gender, we saw an absolute predominance of women, which is logical, but it is worth noting that the EAPC for incidence in men is significantly higher than that in women and continues to increase at an average rate of 0.91% per year. These results suggest that we should not ignore men in the health monitoring of BC in the future, especially for those who have bad behavior factors, such as smoking, alcohol use. The latest study^1^ found that tobacco is the main risk factor of cancer for male, followed by alcohol use, dietary risks and air pollution.

SDI is a composite index calculated based on the total fertility rate of women under the age of 25, the per capita lagged distribution income and the average education level of individuals aged 15 and above^13^. Our results showed that the higher the SDI level, the higher incident cases and ASR of BC, but the EAPC did not appear consistent. On the contrary, in the meddle-high and high SDI regions, the EAPC of BC incidence was significantly reduced, especially in the high SDI region even showed negative growth. These findings were also confirmed by further association analysis, and it was found that EAPC showed a significant negative correlation with ASIR. One possible explanation is that the ASR of BC incidence in these regions was higher in the past, with limited room for increase. Furthermore, due to the general increase in the education level of the population in these areas, people’s awareness of health has been continuously strengthened, which has limited the growth of BC to a certain extent. However, it is worth noting that in low, low-middle, and middle SDI regions, changes in the ASR and EAPC due to population growth and due to the global rise in SDI levels, they are re expected to impose increasing burdens on individuals and societies.

From the analysis of countries or regions, the countries with high ASR of BC incidence 20 years ago were mainly concentrated in high-income countries (such as the United States, New Zealand and the Netherlands), but in 2019, some low-income countries (such as Solomon Islands, Lebanon) rapidly occupy the position of high ASR incidence. These results support our previous hypothesis that low, low-meddle, and middle SDI regions are projected to impose increasing burdens on individuals and society. Furthermore, East Asia is the region with the highest ASR of BC incidence, sub-Saharan Africa is the only region where the EAPC of BC continues to grow, and Solomon Islands is the only country where EAPC has increased by more than 6%. These countries and regions should be the focus of future BC disease burden monitoring.

Further GBD future forecasts for BC demonstrated that that from 2020 to 2050, the global BC incidence and total incidence will increase year by year. Therefore, how to control modifiable risk factors and reduce the incidence of BC becomes the key to alleviating GBD of BC.

### Breast cancer deaths burden

ASR for deaths, as one of the commonly used indicators in disease burden research, can measure the level of risk to the population from the perspective of life^9^. Our study demonstrated that the global death cases of BC in 2019 (700,660 cases) was higher than in 1990 (380,910 cases). There will be 1,503,694 death cases (1,481,463 women and 22,231 men) of BC in the world in 2050. These results suggest that the GBD from breast cancer deaths will remain severe for some time to come. The incidence of BC is dominant in females, so the relatively higher death rate in females may be related to the higher incidence of BC in females than in males. Previous studies^9,11^ have shown that high-income countries such as North America and Western Europe have a higher GBD of BC due to death. Our GBD study based on 1990 also came to a similar conclusion. But what is exciting is that by 2019, the ASR for deaths in high-income countries such as North America and Western Europe has gradually declined the most obvious in all countries. These findings were also confirmed by the results of death cases and death ASR in different SDI index countries and the significant negative association between HDI and EPAC. A logical explanation is that the application in widespread mammography for early-stage BC diagnosis in high-income and high-SDI countries^7,14^ and improved treatment facilities in terms of chemotherapy, radiation therapy, and targeted approaches may be the underlying reasons for the decline in BC mortality in these countries. In addition, our study also found that the growth of EAPC for deaths was particularly rapid in low and low-medium SDI regions and in sub-Saharan Africa. Possible solutions behind this growing trend are access to widespread mammograms, improved BC awareness, increased exercise and greater access to healthcare, among others. It is worth noting that in low-income countries, individuals suffering from severe illnesses may opt to discontinue their treatment because of the considerable financial burden it places on their families. This decision can lead to a rapid deterioration of their condition and ultimately hasten their demise, a phenomenon known as “near-suicide.” Research indicates that as the severity of the illness increases, patients are more likely to forego treatment due to familial responsibilities^15^. These findings underscore the importance of providing accessible and affordable healthcare for individuals dealing with serious illnesses.

### Risk factors attributable to breast cancer burden

The latest research evidence^1^ shown that 44.4% of global cancer deaths and 42.0% of global cancer disability-adjusted life years can be attributed to GBD 2019 estimated risk factors. Our study demonstrates that the major risk factor globally attributable to BC deaths is metabolic risks. High body mass index and high fasting glucose have also been identified as potential risk factors attributable to BC deaths^11^, and our study found that the proportion of contribution of the two risk factors was quite different in various SDI regions of the world. In most countries with high SDI, the growth rate of national wealth is also the fastest, and the growth rate of national wealth is often proportional to the increase in body weight^16^. Our study did not observe a significant increase in the proportion of BC death risk attributable to high body mass index in high-income countries, which may be related to the traditional low-calorie diet^17^ and high physical activity in the part of countries, such as walking^18^. Conversely, the proportion of BC deaths attributable to high body mass index is increasing in low, low-middle SDI regions. It suggests that the body weight control is the key to reduce the risk of BC disease in the future, in these regions, especially in low-middle SDI region. However, glycemic control may be more important for middle and middle-high SDI regions, where a very high proportion of BC deaths are attributable to high fasting glucose.

Alcohol use is one of the important risk factors for BC death^11^, and a pioneering study^19^ has revealed a possible dose-response relationship between alcohol consumption and BC. Our study found that the proportion of disease burden of BC deaths attributable to alcohol use gradually decreased in meddle-high and high SDI countries, which may be very much related to the significant decline in the prevalence of daily alcohol consumption globally^20^. These results may also better explain why the incidence and deaths of BC in high-income countries such as North America and Europe have gradually decreased, despite high-calorie, high-metabolic diets.

The GBD 2019 Study shows that smoking remains the leading cause of cancer death and health loss worldwide^1^. Our study found that the proportion of BC disease burden attributable to smoking decreased gradually over time in low, low- middle, and middle SDI regions, which may be related to the decline in smoking prevalence in these regions. Because previous study^21^ has shown that smoking rates decline with the lower SDI. In addition, our study indicates that the dietary risk and low physical activity also play a role in the burden of BC disease. Therefore, reducing the global burden of breast cancer requires a comprehensive cancer prevention and control strategy. On the one hand, reduce the incidence and death of breast cancer by controlling adverse metabolic risks and behavioral risks (such as alcohol use, tobacco, dietary risk, low physical activity); on the other hand, by promoting mammography for early diagnosis of breast cancer, and improvements in effective treatments to effectively reduce the global burden of disease.

### Strengths and limitations

To our knowledge, this GBD-based study is the largest effort to date to reveal global BC incidence and deaths, determine the global cancer burden attributable to the most relevant risk factors, and predict the future burden of BC. The study will help to enrich the research evidence of global BC risk and attributable disease burden^11,22–25^, which is of great important to prevent and control the occurrence and development of BC and improve health. However, our study also has limitations. First, some countries (or territories) do not have population-based cancer registries, leaving an important source of data for estimating cancer burden missing. Second, the GBD2019 only provides some behavioral risks (such as tobacco, alcohol use, dietary risks and low physical activity) and metabolic risks (including high fasting glucose and high body mass index) that can be used for further research^26^, are important for a comprehensive assessment of the burden of breast cancer attributable to risk factors. Finally, the disability-adjusted life years, as an index that can simultaneously consider premature death from disease and health loss from disability, has received increasing attention in the field of international cancer disease burden evaluation^27^, and this study focuses on diseases caused by BC incidence and deaths burden.

Moreover, data sharing, a practice that enhances research integrity and transparency, facilitating peer validation and enabling further exploration of the study’s findings, offers valuable resources for scientific research and evidence-based policymaking, particularly relevant to developing countries^28,29^. In this sense, our research has significant meaning, because all data available free of charge. Our data provide a rigorous and comparable measure of the global disease burden of breast cancer, all freely downloadable, and can be used by policymakers in the future to generate the evidence they need on how to allocate resources to best improve the population Health makes informed decisions. Nonetheless, it is worth noting that our findings may be delayed as they reflect past disease burden. While our analysis provides valuable insights into the historical trends of breast cancer, predicting future trends necessitates confirmation by more recent data.

Overall, our study provides some evidence that regions with low levels of SDI will have the largest disease burden of breast cancer in the future. Metabolic risk factors increased the most from 1990 to 2019, compared with the behavioral factors. The findings of this study may be of great value for preventing and controlling the incidence and deaths of BC, as well as for improving the health of population. Furthermore, our study results may aid decision makers in formulating more reasonable and effective preventive health policies, and solutions for BC, and related health inequalities.

## Methods

### Study design

In this study covering data of GBD on incidence, deaths, and their temporal trends in 204 countries or territories and 21 regions from 1990 to 2019, different changing trends of BC burden were observed, with significant differences by sex, region, country, and sociodemographic index. The logical flowchart of this study is shown in Supplementary Fig. 4.

### Data sources

Annual incident cases, age standardized incidences and deaths of BC from 1990 to 2019, by sex, region, country, and risk factors (metabolic risks, dietary risks, tobacco, alcohol use and low physical activity) were obtained from the GBD 2019 through the Global Health Data Exchange (GHDx) query tool (https://ghdx.healthdata.org/gbd-2019).

To create the source dataset, we follow a procedure. First, we access the data acquisition interface of the database and click on the “query tool” hyperlink located under the “GBD Results Tool” menu. This leads us to the data retrieval interface where we have the option to select different GBD evaluation options from the “GBD Estimate” drop-down menu. By default, cause of death or injury is selected. Next, we can choose from a variety of disease evaluation indicators such as morbidity, prevalence, mortality, and disease burden, including disability-adjusted life years, from the “Measure” drop-down menu. We can also select different measurement indicators like number, percent, and rate from the “Metric” drop-down menu. The “Cause” drop-down menu provides an extensive list of common causes such as tumor, high blood pressure, and diabetes, among others. Similarly, the “Location” drop-down menu displays all the countries and regions in the world which are categorized in great detail, including China (national level) and East Asia (regional level). For some countries like the United States and the United Kingdom, intra-country state or provincial level data is also available although this feature is not currently available for China. Moreover, we can filter data by age and sex using the “Age and Sex” drop-down menu, while the “Year” drop-down menu allows us to choose a time range between 1990–2019. GBD 2019 is the most up to date and ongoing global collaboration, and all epidemiological data are available as open source. Simply enter your desired query in the search box above and click “Search” to retrieve the relevant information. Alternatively, you may choose to directly download the CSV file by clicking on the “Download CSV” button.

### Data records

A total of 204 countries or territories and 21 regions were selected in this study. The human development index (HDI) data at the national level were collected from the United Nations Development Programme (https://hdr.undp.org/data-center/human-development-index#/indicies/HDI). Rates in this study are reported per 100,000 people, and age-standardized rates are calculated based on GBD world population standards^30^. Some of the results were provided by the sociodemographic index (SDI) to describe differences in GBD of BC. The quintiles of the SDI index are used to define low (~20), low-middle (~40), middle (~60), middle-high (~80) and high (~100) SDI countries in 2019^27^. The global population forecast data for 2017–2100 were obtained from the Institute for Health Metrics and Evaluation (https://ghdx.healthdata.org/record/ihme-data/global-population-forecasts-2017-2100). The data supports this finding have recorded in the Figshare^31^ (10.6084/m9.figshare.22787405). The document “GBD for BC.xlsx” comprises six main worksheets. The first worksheet, named “BC_nation,” is primarily utilized to analyze the country’s morbidity and mortality related to BC, enabling quantification of this data. The following worksheets - “BC_region,” “BC_region_SDI,” and “BC_region_SEX” - are used to examine the morbidity and mortality of BC quantified by region, SDI, and sex. To assess trends in BC incidence and mortality, an estimation of the change in cancer cases from 1990 to 2019, along with the EAPC and its 95% confidence interval, are used. Finally, the “BC_percent” worksheet focuses on estimating the cancer burden attributable to risk factors.

### Data analysis

Referring to previous literature report^32^, the age-standardized ratio (ASR) and its 95% uncertainty interval was used to quantify the incidence and deaths of BC by time, sex, region, country and SDI. Then, the changes in cancer cases, the estimated annual percentage change (EAPC) and its 95% confidence interval from 1990 to 2019 was used to assess the incidences and deaths trend of BC. Finally, we combined EAPC data for incidences and deaths to perform hierarchical cluster analysis to identify countries with similar annual increases in incidences and deaths. All countries were divided into 4 groups, including minor increase, remained stable or minor decrease, significant decrease, and significant increase.

GBD 2019 includes three categories of attributable risks, such as environment or occupation risks, behavior risks and metabolism risks. We first identified the BC risk factors with convincing or likely causal evidence based on World Cancer Research Fund criteria. Then, the proportion of cancer-specific burden attributable to each risk factor was calculated in different year, region, country and SDI. Finally, temporal trends of attributable risk factors were assessed at the SDI level.

We selected two-time nodes, 1990 and 2019, and calculated the age-standardized incidence rates (ASIR) and age-standardized deaths rates (ASDR) at the country level. Then, HDI, ASIR, ASDR were selected as the candidate indicators to determine the influencing factors of EAPC by correlation analysis.

Considering that the incidence and mortality rates of different sexes are different, in this study we separately predicted the incidence and deaths rates of men and women from 2020 to 2050 to assess the future GBD of BC. This GBD forecasts is primarily based on the Global Population Forecasts 2017–2100 data and age-standardized BC incidence and deaths data from 1990 to 2019.

All statistical analysis of data were performed using the R Project for Statistical Computing (version 4.2.2; R Core Team). We used the ASR and EAPC to quantify the BC incidence and deaths trends. Constituent ratios were used to evaluate the cancer burden attributable to risk factors. Pearson correlation analysis was used to determine the association of HDI, ASIR, ASDR with EAPC. For the future forecasts of GBD in BC, we used the BACP package. A threshold of P value less than 0.05 was set to determine the significant differences.

## Usage Notes

Our data and code are freely available as open source. The analysis codes presented in the article were written using the R language. To conduct your own analysis, you will need to first install the necessary environment for R, including packages such as dplyr, ggplot2, ggsci, factoextra, ggmap, rgdal, maps, devtools, and others. Moving forward, these data can be utilized to examine the disease burden of breast cancer and its changing trends, categorized by time, sex, region, country, and SDI. Additionally, if you intend to analyze other disease burdens apart from breast cancer, our open-source R language code is also well-suited for your needs.

## Supplementary information

Supplementary Information

## Data Availability

The data supports this finding can be accessed from the Figshare^31^ (10.6084/m9.figshare.22787405). The “Date.xlsx” file contains separate sheets that provide pertinent metadata for assessing the incidence and mortality rates of breast cancer based on various factors such as time, gender, region, country, and socio-demographic index (SDI). In addition to this information, the document also includes data on the World population age standard, the HDI of different countries in 1990, and Global Population Forecasts spanning from 2017 to 2100.

## Code availability

All R code supporting the conclusions of this study can be accessed and downloaded via Github^33^ (10.5281/zenodo.7915783). The main computational tools used in this study are R language based. The scripts used in this study include “GBD_Incidence_region.R,” which calculates incident cases, deaths, ASR, and EAPC for BC worldwide during 1990 and 2019. Another script, “GBD_Incidence_map.R” is employed to generate visualizations of BC incidence and mortality using GBD data from 204 countries and regions around the world. Additionally, “GBD_cluster.R” was used to perform hierarchical cluster analysis to identify countries with similar annual increases in BC incidence and mortality. To calculate the percentage of major risk factors globally attributable to BC mortality, “GBD_Percent.R” was utilized. The correlation between EAPC and ASIR, ASDR, and HDI was analyzed using “GBD_COR.R”. Finally, “Global_BAPC_prediction.R” was implemented to predict the future burden of BC using GBD data.
