# Supplementary material for: Global trends and forecasts of breast cancer incidence and deaths
Source: Sci Data. 2023 May 27;10:334. doi: 10.1038/s41597-023-02253-5 (PMC10224917; doi:10.1038/s41597-023-02253-5)
Supplement: Supplementary file 1 — Supplementary Information [file 41597_2023_2253_MOESM1_ESM.docx]

**Supplementary Information**

*Global trends and forecasts of breast cancer incidence and deaths*

*Xu et al.*

**Content**

[Supplementary Table 1. The incident cases and age-standardized incidence of breast cancer in 1990 and 2019, and its temporal trends from 1990 to 2019 1](#_Toc135165769)

[Supplementary Table 2. The death cases and age-standardized death of breast cancer in 1990 and 2019, and its temporal trends from 1990 to 2019 3](#_Toc135165770)

[Supplementary Fig. 1. The cluster dendrogram based on EAPC incidence and mortality in the countries or territories. 5](#_Toc135165772)

[Supplementary Fig. 2. The GBD of BC attributable to risk factors in different SDI regions and yeas. 6](#_Toc135165774)

[Supplementary Fig. 3. The GBD of BC attributable to metabolism risks. 7](#_Toc135165776)

[Supplementary Fig. 4. The logical flowchart of this study. 8](#_Toc135165778)

# Supplementary Table 1. The incident cases and age-standardized incidence of breast cancer in 1990 and 2019, and its temporal trends from 1990 to 2019

| **Characteristics** | **1990** |  | |  | **2019** |  | |  | **1990-2019** |
| --- | --- | --- | --- | --- | --- | --- | --- | --- | --- |
|  | **Incident cases** | | **ASR per 100,000** |  | **Incident cases** | | **ASR per 100,000** |  | **EAPC** |
|  | **No. ×10^3^ (95% UI)** | | **No. (95% UI)** |  | **No. ×10^3^ (95% UI)** | | **No. (95% UI)** |  | **No. (95% CI)** |
| **Overall** | 876.99(849.69-903.82) | | 21.44 (20.65-22.10) |  | 2002.35(1832.15-2172.54) | | 24.17 (22.11-26.24) |  | 0.33 (0.28-0.37) |
| **Sex** |  | |  |  |  | |  |  |  |
| Female | 867.62(840.40-894.76) | | 40.12 (38.78-41.33) |  | 1977.21(1807.61-2145.21) | | 45.86 (41.91-49.76) |  | 0.36 (0.31-0.42) |
| Male | 9.37(8.81-9.96) | | 0.53 (0.5-0.57) |  | 25.14(22.23-27.79) | | 0.65 (0.58-0.72) |  | 0.91 (0.76-1.06) |
| **Socio-demographic index** |  | |  |  |  | |  |  |  |
| Low SDI | 24.85(20.72-28.76) | | 9.24 (7.66-10.79) |  | 81.72(71.26-93.26) | | 13.51 (11.93-15.22) |  | 1.28 (1.22-1.34) |
| Low-middle SDI | 66.10(58.24-74.33) | | 9.58 (8.39-10.74) |  | 230.77(202.83-259.30) | | 15.38 (13.54-17.27) |  | 1.54 (1.44-1.64) |
| Middle SDI | 125.97(116.28-136.06) | | 10.66 (9.87-11.48) |  | 493.15(437.19-552.89) | | 18.52 (16.43-20.76) |  | 1.95 (1.92-1.98) |
| Middle-High SDI | 228.47(220.96-236.31) | | 20.93 (20.23-21.63) |  | 516.51(464.37-574.12) | | 26 (23.34-28.88) |  | 0.68 (0.59-0.77) |
| High SDI | 431.08(416.85-440.22) | | 43.2 (41.83-44.11) |  | 678.94(606.88-753.70) | | 41.22 (36.88-45.65) |  | -0.27 (-0.37--0.18) |
| **Region** |  | |  |  |  | |  |  |  |
| High-income Asia Pacific | 13.20(12.65-13.57) | | 6.50 (6.23-6.69) |  | 25.16(21.21-29.62) | | 5.71 (4.83-6.76) |  | -0.57 (-0.74--0.40) |
| Central Asia | 6.41(6.21-6.65) | | 13.80 (13.32-14.34) |  | 4.83(4.27-5.68) | | 6.70(5.95-7.75) |  | -2.85 (-3.08--2.61) |
| East Asia | 176.24(114.35-205.92) | | 20.48 (13.39-23.74) |  | 284.91(220.17-338.89) | | 13.72 (10.64-16.25) |  | -1.54 (-1.98--1.09) |
| South Asia | 25.60(22.50-32.08) | | 4.52 (3.99-5.67) |  | 53.49(46.15-72.05) | | 3.78 (3.27-5.10) |  | -0.86 (-0.98--0.73) |
| Southeast Asia | 7.09(6.04-8.06) | | 2.75 (2.37-3.11) |  | 15.54(13.19-18.20) | | 2.54 (2.18-2.97) |  | -0.34 (-0.39--0.30) |
| Australasia | 1.08(1.02-1.12) | | 4.58 (4.36-4.77) |  | 2.19(1.77-2.71) | | 4.41 (3.55-5.46) |  | -0.30 (-0.39--0.20) |
| Caribbean | 0.98(0.92-1.06) | | 3.83 (3.59-4.14) |  | 1.92(1.64-2.20) | | 3.69 (3.15-4.23) |  | 0.07 (-0.13-0.27) |
| Central Europe | 4.28(4.16-4.39) | | 2.90 (2.81-2.97) |  | 5.85(5.11-6.66) | | 2.89 (2.52-3.3) |  | -0.17 (-0.27--0.07) |
| Eastern Europe | 12.16(11.70-12.87) | | 4.27 (4.1-4.53) |  | 11.09(9.67-12.60) | | 3.25 (2.83-3.69) |  | -1.38 (-1.62--1.14) |
| Western Europe | 27.00(26.24-27.50) | | 4.84 (4.71-4.92) |  | 40.17(35.13-45.71) | | 4.64 (4.06-5.29) |  | -0.23 (-0.34--0.12) |
| Andean Latin America | 0.39(0.33-0.44) | | 1.95 (1.64-2.20) |  | 0.83(0.67-1.02) | | 1.51 (1.22-1.85) |  | -0.85 (-0.94--0.76) |
| Central Latin America | 1.91(1.84-1.97) | | 2.39 (2.28-2.47) |  | 3.87(3.28-4.51) | | 1.66 (1.41-1.94) |  | -1.48 (-1.6--1.37) |
| Southern Latin America | 3.38(3.26-3.50) | | 7.43 (7.15-7.68) |  | 3.94(3.16-4.94) | | 4.70 (3.75-5.89) |  | -1.93 (-2.09--1.77) |
| Tropical Latin America | 6.13(5.91-6.37) | | 6.66 (6.39-6.93) |  | 12.68(11.99-13.29) | | 5.17 (4.87-5.42) |  | -0.84 (-0.89--0.79) |
| North Africa and Middle East | 4.37(3.08-5.24) | | 2.54 (1.85-3.03) |  | 10.02(7.41-11.44) | | 2.36 (1.79-2.66) |  | -0.32 (-0.36--0.28) |
| High-income North America | 13.20(12.78-13.56) | | 3.86 (3.75-3.96) |  | 26.16(22.46-30.59) | | 4.22 (3.63-4.96) |  | 0.19 (0.07-0.31) |
| Oceania | 0.06(0.05-0.09) | | 2.18 (1.69-3.06) |  | 0.15(0.11-0.20) | | 2.15 (1.66-2.89) |  | -0.05 (-0.08--0.03) |
| Central Sub-Saharan Africa | 2.48(1.15-3.30) | | 10.84 (5.04-14.27) |  | 4.43(2.38-6.02) | | 8.41 (4.48-11.59) |  | -1.09 (-1.18--1.00) |
| Eastern Sub-Saharan Africa | 8.44(6.39-9.88) | | 11.18 (8.49-13.03) |  | 16.39(12.43-20.71) | | 10.03 (7.71-12.6) |  | -0.46 (-0.54--0.38) |
| Southern Sub-Saharan Africa | 3.72(2.79-4.58) | | 13.31 (9.93-16.49) |  | 5.94(5.32-6.94) | | 10.66 (9.56-12.29) |  | -1.36 (-1.89--0.81) |
| Western Sub-Saharan Africa | 1.84(1.58-2.14) | | 2.10 (1.82-2.44) |  | 4.99(3.78-5.99) | | 2.71 (2.06-3.21) |  | 1.16 (1.04-1.28) |

ASR, age standardized rate; CI, confidence interval; EAPC, estimated annual percentage change; UI, uncertainty interval; SDI, socio-demographic index.

# Supplementary Table 2. The death cases and age-standardized death of breast cancer in 1990 and 2019, and its temporal trends from 1990 to 2019

| **Characteristics** | **1990** |  | |  | **2019** |  | |  | **1990-2019** |
| --- | --- | --- | --- | --- | --- | --- | --- | --- | --- |
|  | **Death cases** | | **ASR per 100,000** |  | **Death cases** | | **ASR per 100,000** |  | **EAPC** |
|  | **No. ×10^3^ (95% UI)** | | **No. (95% UI)** |  | **No. ×10^3^ (95% UI)** | | **No. (95% UI)** |  | **No. (95% CI)** |
| **Overall** | 380.91(364.81-396.71) | | 9.8(9.3-10.21) |  | 700.66(647.38-751.56) | | 8.62 (7.95-9.25) |  | -0.56 (-0.6--0.51) |
| **Sex** |  | |  |  |  | |  |  |  |
| Female | 375.02(358.98-390.82) | | 17.76(16.93-18.51) |  | 688.56(635.32-739.57) | | 15.88 (14.66-17.07) |  | -0.51 (-0.56--0.46) |
| Male | 5.89(5.43-6.37) | | 0.37(0.34-0.4) |  | 12.10(10.69-13.32) | | 0.33 (0.29-0.36) |  | -0.23 (-0.36--0.1) |
| **Socio-demographic index** |  | |  |  |  | |  |  |  |
| Low SDI | 19.51(16.31-22.90) | | 7.79(6.47-9.3) |  | 54.47(47.49-62.06) | | 9.83 (8.59-11.12) |  | 0.75 (0.71-0.8) |
| Low-middle SDI | 46.52(40.56-52.30) | | 7.23(6.24-8.14) |  | 127.41(110.47-144.92) | | 8.94 (7.77-10.16) |  | 0.63 (0.54-0.72) |
| Middle SDI | 72.64(67.62-78.67) | | 6.67(6.2-7.17) |  | 184.79(166.33-205.34) | | 7.3 (6.6-8.12) |  | 0.29 (0.26-0.32) |
| Middle-High SDI | 104.54(100.73-108.32) | | 9.98(9.58-10.35) |  | 165.93(152.74-179.59) | | 8.31 (7.63-9) |  | -0.83 (-0.95--0.71) |
| High SDI | 137.44(130.74-140.80) | | 13.55(12.9-13.88) |  | 167.55(151.81-176.81) | | 9.05 (8.36-9.47) |  | -1.52 (-1.56--1.47) |
| **Region** |  | |  |  |  | |  |  |  |
| High-income Asia Pacific | 9.60(9.20-9.83) | | 4.79(4.56-4.92) |  | 20.69(17.89-22.51) | | 5.18 (4.68-5.53) |  | 0.45 (0.32-0.59) |
| Central Asia | 5.22(5.03-5.41) | | 10.83(10.42-11.21) |  | 7.58(6.79-8.46) | | 9.85 (8.87-10.89) |  | -0.22 (-0.31--0.13) |
| East Asia | 43.70(36.39-51.42) | | 4.77(3.99-5.57) |  | 101.02(82.36-122.87) | | 4.91 (3.99-5.94) |  | 0.09 (0.03-0.14) |
| South Asia | 41.22(33.88-47.37) | | 6.82(5.52-7.95) |  | 127.80(105.47-151.68) | | 8.72 (7.23-10.31) |  | 0.70 (0.59-0.81) |
| Southeast Asia | 29.25(26.42-33.31) | | 10.14(9.22-11.42) |  | 67.28(57.79-77.24) | | 10.38 (8.98-11.85) |  | -0.01 (-0.07-0.06) |
| Australasia | 3.26(3.10-3.37) | | 14.46(13.7-14.94) |  | 4.48(4.03-4.83) | | 9.36 (8.60-10.03) |  | -1.66 (-1.77--1.56) |
| Caribbean | 2.77(2.61-2.93) | | 10.62(10.01-11.20) |  | 5.80(4.94-6.79) | | 11.22 (9.53-13.11) |  | 0.32 (0.24-0.40) |
| Central Europe | 17.58(17.06-18.02) | | 12.39(11.95-12.72) |  | 23.35(20.39-26.57) | | 11.32 (9.86-12.93) |  | -0.34 (-0.44--0.25) |
| Eastern Europe | 30.01(29.19-30.96) | | 11.00(10.68-11.36) |  | 35.37(30.68-40.86) | | 10.59 (9.17-12.27) |  | -0.6 (-0.87--0.32) |
| Western Europe | 88.95(84.54-91.16) | | 16.21(15.43-16.59) |  | 98.41(88.19-104.25) | | 10.9 (10.01-11.45) |  | -1.54 (-1.6--1.48) |
| Andean Latin America | 1.46(1.31-1.63) | | 6.67(6-7.44) |  | 3.82(3.14-4.69) | | 6.69 (5.52-8.18) |  | -0.18 (-0.29--0.07) |
| Central Latin America | 5.82(5.64-5.95) | | 6.49(6.24-6.66) |  | 16.83(14.45-19.74) | | 6.98 (6.00-8.18) |  | 0.24 (0.17-0.30) |
| Southern Latin America | 7.24(6.97-7.48) | | 16.09(15.42-16.65) |  | 11.24(10.40-12.01) | | 13.6 (12.62-14.52) |  | -0.72 (-0.84--0.60) |
| Tropical Latin America | 9.04(8.71-9.35) | | 9.59(9.14-9.94) |  | 20.66(19.27-21.90) | | 8.46 (7.87-8.97) |  | -0.49 (-0.64--0.33) |
| North Africa and Middle East | 11.92(10.79-13.69) | | 6.34(5.72-7.30) |  | 36.21(31.42-41.54) | | 7.62 (6.66-8.68) |  | 0.68 (0.61-0.75) |
| High-income North America | 52.89(50.22-54.31) | | 15.52(14.82-15.91) |  | 61.59(56.94-64.84) | | 10.04 (9.39-10.51) |  | -1.7 (-1.79--1.61) |
| Oceania | 0.57(0.45-0.71) | | 16.13(12.91-19.65) |  | 1.81(1.39-2.30) | | 21.04 (16.32-26.53) |  | 0.93 (0.89-0.97) |
| Central Sub-Saharan Africa | 2.33(1.86-2.84) | | 9.85(8.04-11.83) |  | 6.99(5.08-9.18) | | 12.87 (9.37-16.91) |  | 0.81 (0.71-0.92) |
| Eastern Sub-Saharan Africa | 7.11(5.99-8.28) | | 9.26(7.73-10.87) |  | 17.82(15.24-20.47) | | 10.74 (9.29-12.13) |  | 0.45 (0.35-0.55) |
| Southern Sub-Saharan Africa | 3.00(2.68-3.39) | | 10.78(9.49-12.40) |  | 7.26(6.45-8.09) | | 13.33 (12.02-14.77) |  | 1.03 (0.80-1.26) |
| Western Sub-Saharan Africa | 7.98(6.49-9.98) | | 8.83(7.15-11.02) |  | 24.64(19.34-30.97) | | 12.27 (9.85-15.09) |  | 1.20 (1.09-1.30) |

ASR, age standardized rate; CI, confidence interval; EAPC, estimated annual percentage change; UI, uncertainty interval; SDI, socio-demographic index.

###
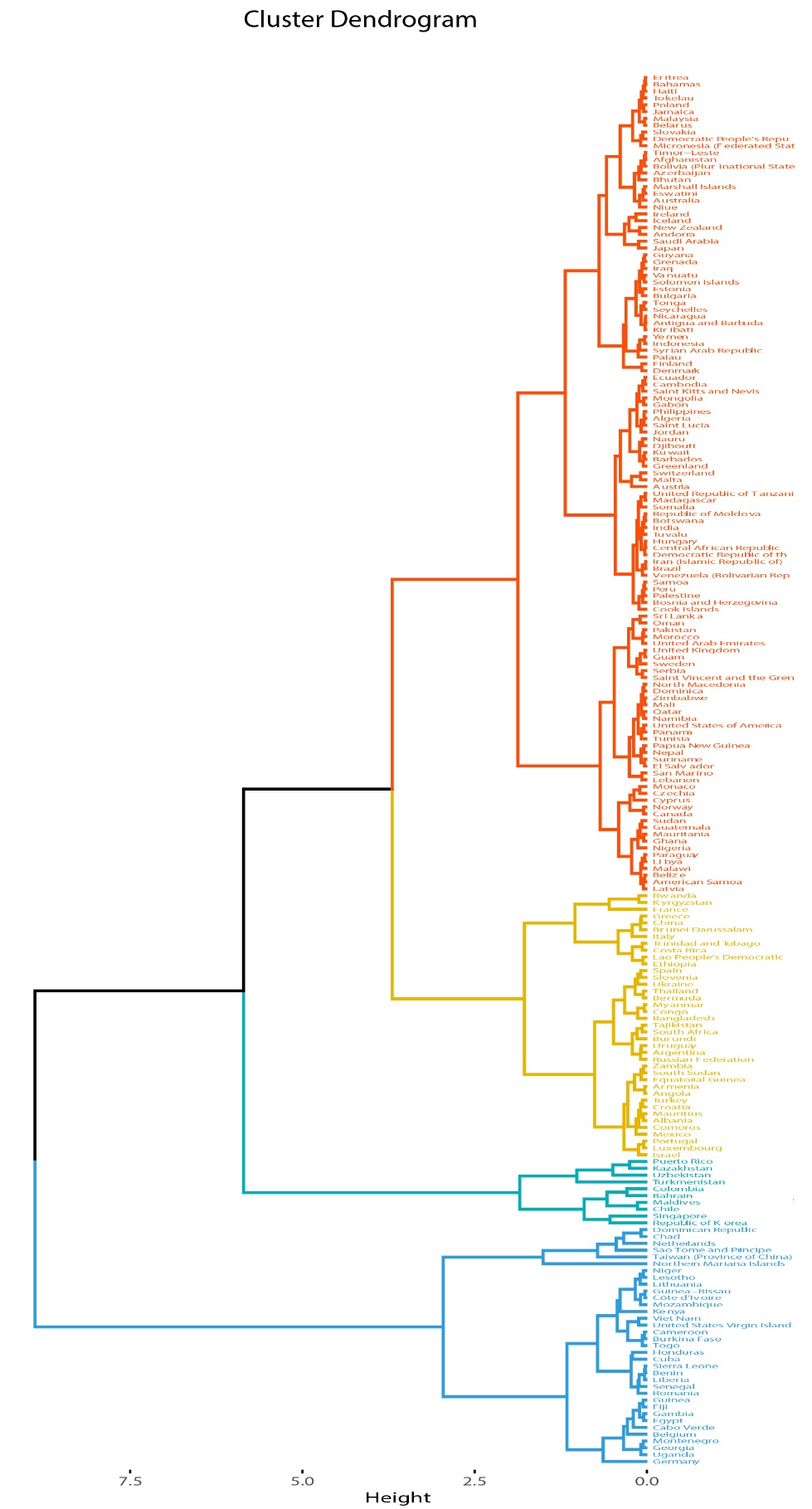


# Supplementary Fig. 1. The cluster dendrogram based on EAPC incidence and mortality in the countries or territories.

###
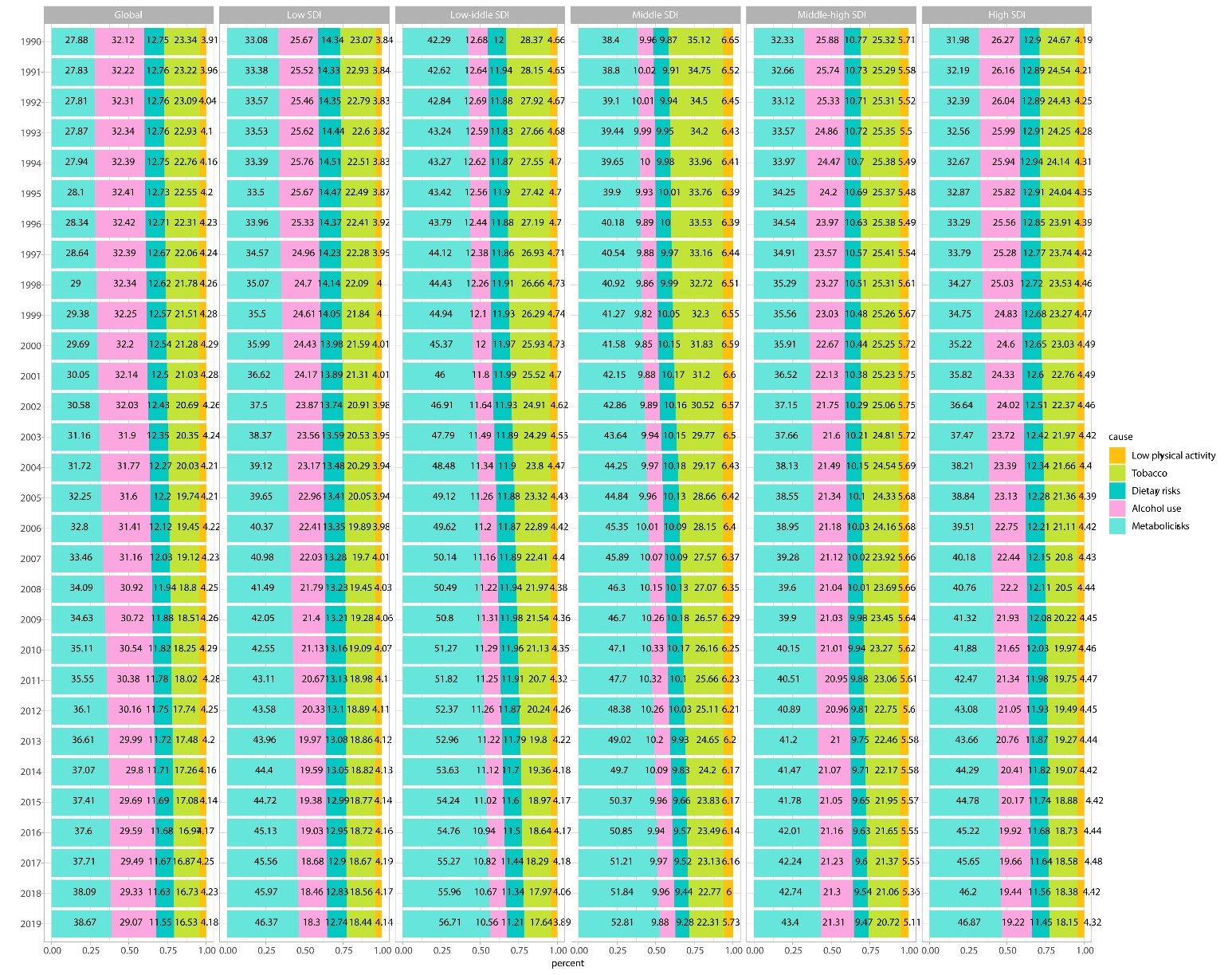


# Supplementary Fig. 2. The GBD of BC attributable to risk factors in different SDI regions and yeas.

###
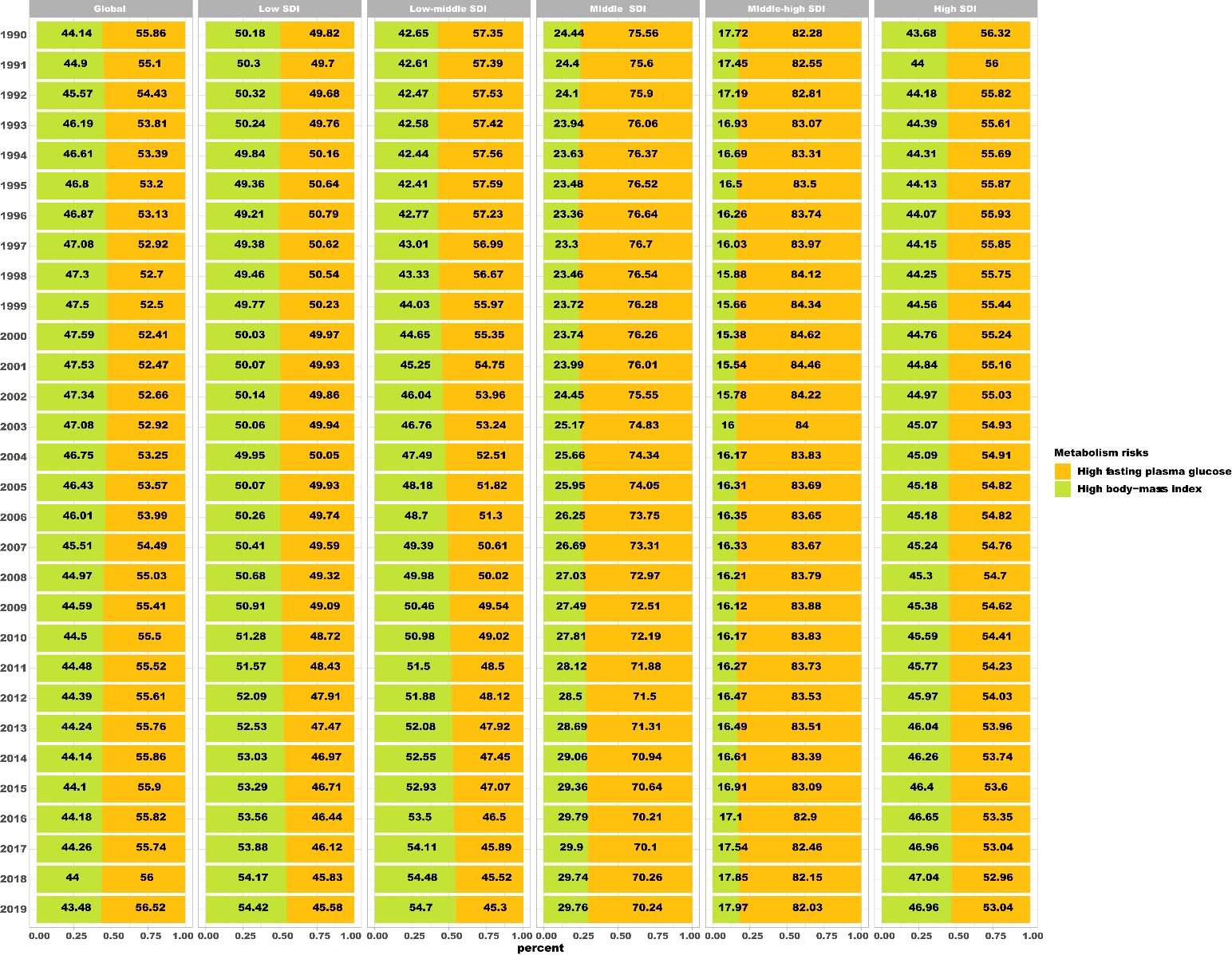


# Supplementary Fig. 3. The GBD of BC attributable to metabolism risks.

###
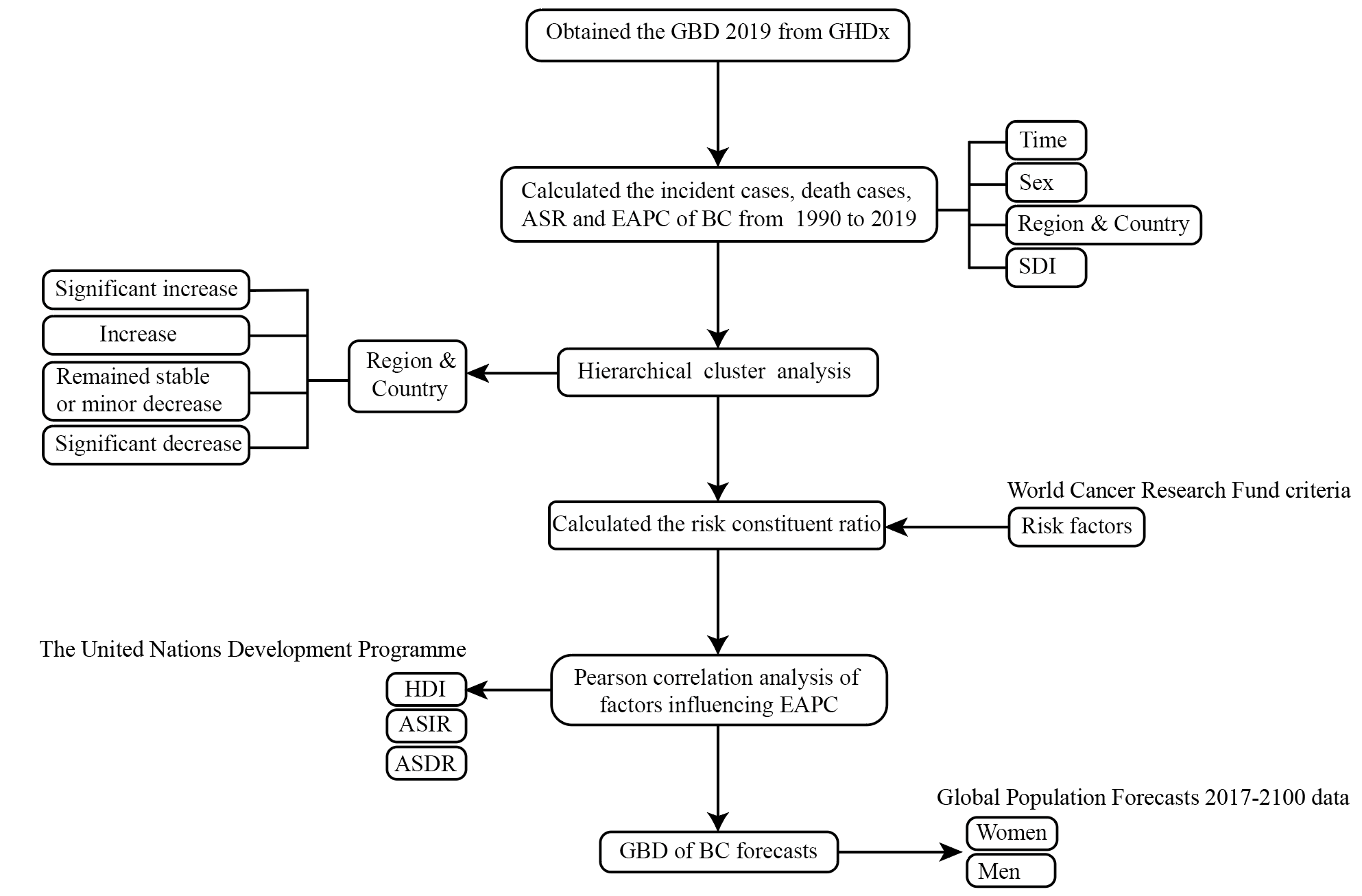


# Supplementary Fig. 4. The logical flowchart of this study.
